# Supplementary material for: The interactome of intact mitochondria by cross-linking mass spectrometry provides evidence for coexisting respiratory supercomplexes
Source: Mol Cell Proteomics. 2017 Dec 8;17(2):216–32. doi: 10.1074/mcp.RA117.000470 (PMC5795388; doi:10.1074/mcp.RA117.000470)
Supplement: Supplemental Data [file supp_RA117.000470_133922_0_supp_23978_fzffwf.zip › spectra_annotation/mito_spectra_annotation/341-1-37-1-1-1.pdf]

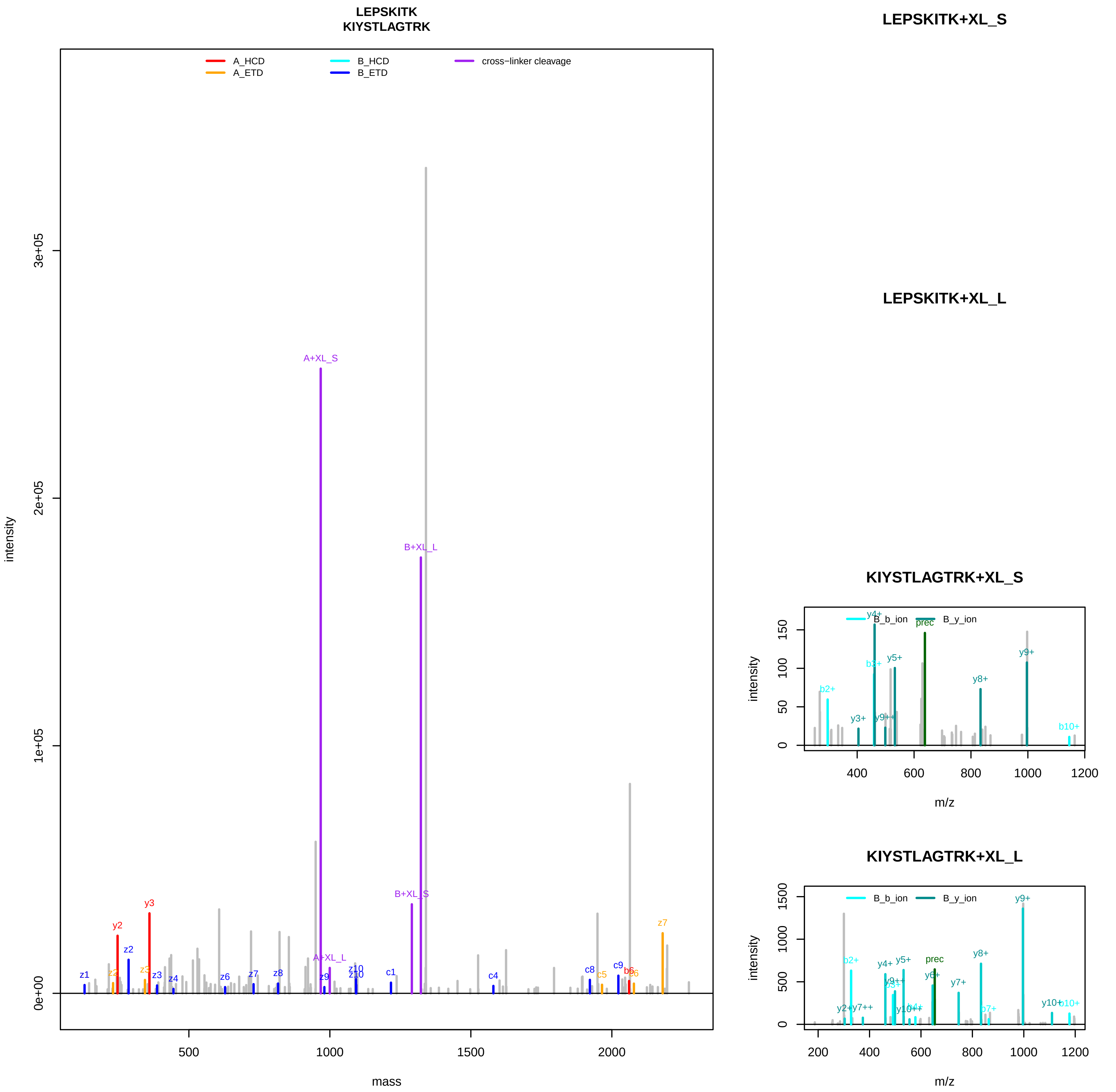
LEPSKITK
KIYSTLAGTRK
A_HCD
A_ETD
B_HCD
B_ETD
cross−linker cleavage
A+XL_S
B+XL_L
B+XL_S
A+XL_L
y2
y3
b6
z1
z2
z3
z4
z6
z7
z8
z9
z10
c1
c4
c5
c6
c8
c9
z7
intensity
mass
0e+00
1e+05
2e+05
3e+05
500
1000
1500
2000
LEPSKITK+XL_S
LEPSKITK+XL_L
KIYSTLAGTRK+XL_S
B_b_ion
B_y_ion
prec
b2+
b3+
b10+
y3+
y4+
y5+
y8+
y9+
y9++
m/z
400
600
800
1000
1200
0
50
100
150
KIYSTLAGTRK+XL_L
y2+
y7+
y7++
y6+
y10+
y10++
b4+
b7+
200
500
1000
1500
